# Supplementary material for: The Small RNA Universe of Capitella teleta
Source: Front Mol Biosci. 2022 Feb 25;9:802814. doi: 10.3389/fmolb.2022.802814 (PMC8915122; doi:10.3389/fmolb.2022.802814)
Supplement: Supplementary file 1 [file DataSheet1.ZIP › Supplement/known/CAPTEscaffold_70_6991.pdf]

Provisional ID	:	CAPTEscaffold_70_6991
Score total	:	7176742.2
Score for star read(s)	:	3.9
Score for read counts	:	7176732.9
Score for mfe	:	0.8
Score for randfold	:	1.6
Score for cons. seed	:	3
Total read count	:	14076864
Mature read count	:	14076781
Loop read count	:	3
Star read count	:	80

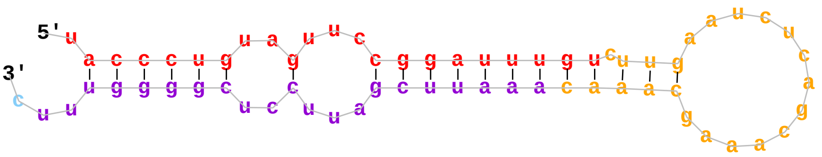

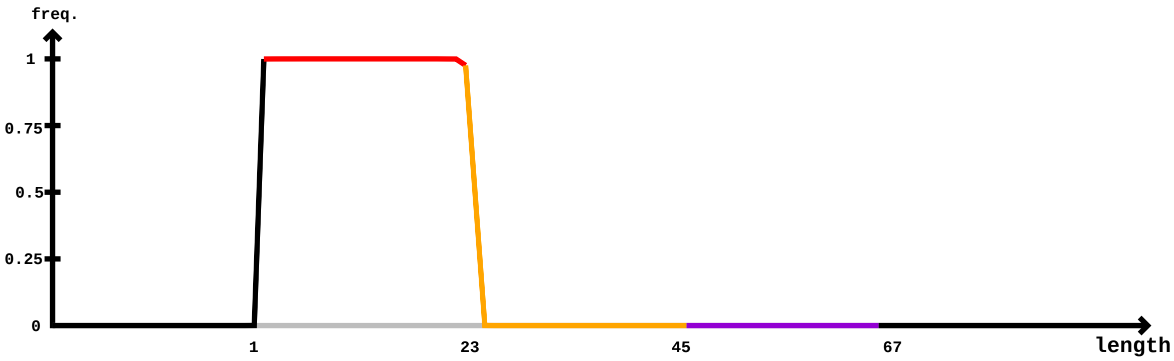


```
                     Mature                                   Star
5'- cuucagugcuccucaauguuuacccuguaguuccggauuugucuugaaucucagcaaagcaaacaaauucgauuccucgggguuucauguugagugagacuuaucuucuagg   -3'   obs
    cuucagugcuccucaauguuuacccuguaguuccggauuugucuugaaucucagcaaagcaaacaaauucgauuccucgggguuucauguugagugagacuuaucuucuagg         exp
    cuucagugcuccucaauguuuacccuguaguuccggauuugucuugaaucucagcaaagcaaacaaauucgauuccucgggguuucauguugagugagacuuaucuucuagg         known
    ....(((.(((((((((((..((((((..(...(((((((((.(((.............))))))))))))...)..))))))...)))))))).))))))...........    reads     mm     sample
    .................Auuuacccuguaguuccggauuugu......................................................................    1         1      seq
    .................guCuacccuguaguuccggauuugu......................................................................    1         1      seq
    .................gAuuacccuguaguuccggauuugu......................................................................    1         1      seq
    ..................uCuacccuguaguuccggauuug.......................................................................    17        1      seq
    ..................uCuacccuguaguuccggauuugu......................................................................    630       1      seq
    ...................Cuacccuguaguuccggau..........................................................................    82        1      seq
    ...................Cuacccuguaguuccggauu.........................................................................    2         1      seq
    ...................Cuacccuguaguuccggauuug.......................................................................    134       1      seq
    ...................uuacccuguaguuccggauuugu......................................................................    21        0      seq
    ...................Cuacccuguaguuccggauuugu......................................................................    3917      1      seq
    ...................Auacccuguaguuccggauuugu......................................................................    14        1      seq
    ....................uacccuguaguGccggau..........................................................................    1         1      seq
    ....................Aacccuguaguuccggau..........................................................................    1         1      seq
    ....................uacccuguaguuccggaA..........................................................................    57        1      seq
    ....................uacccuguaguuccggUu..........................................................................    1         1      seq
    ....................uacccuguaguAccggau..........................................................................    1         1      seq
    ....................uacccuguagAuccggau..........................................................................    1         1      seq
    ....................uacccuguaguuccggau..........................................................................    261       0      seq
    ....................uacccuguaguuccggGu..........................................................................    2         1      seq
    ....................uacccuguaguuccggaCu.........................................................................    1         1      seq
    ....................uacccuguaguuccggGuu.........................................................................    1         1      seq
    ....................uacccuguaAuuccggauu.........................................................................    1         1      seq
    ....................uacccuguaguuccggaAu.........................................................................    4         1      seq
    ....................uacccuguaguuccggauC.........................................................................    3         1      seq
    ....................uacccugCaguuccggauu.........................................................................    2         1      seq
    ....................uacccuguaguuccgAauu.........................................................................    2         1      seq
    ....................uacccAguaguuccggauu.........................................................................    1         1      seq
    ....................uacccuguaguuGcggauu.........................................................................    1         1      seq
    ....................uacccuguaguuUcggauu.........................................................................    1         1      seq
    ....................uacccuguaguuccggauu.........................................................................    3271      0      seq
    ....................uacccuguaguuAcggauu.........................................................................    4         1      seq
    ....................uacccuguaguucUggauu.........................................................................    2         1      seq
```

```
                    Mature                                    Star
cuucagugcuccucaauguuuacccuguaguuccggauuugucuugaaucucagcaaagcaaacaaauucgauuccucgggguuucauguugagugagacuuaucuucuagg
....................Nacccuguaguuccggauu.........................................................................   1        1          seq
....................uacUcuguaguuccggauu.........................................................................   2        1          seq
....................uaAccuguaguuccggauu.........................................................................   2        1          seq
....................uacAcuguaguuccggauu.........................................................................   2        1          seq
....................uacccuguaguuccCgauu.........................................................................   1        1          seq
....................uacccuguaguuccUgauu.........................................................................   2        1          seq
....................uacccuguaguuccggaGu.........................................................................   1        1          seq
....................uacccuguaguAccggauu.........................................................................   1        1          seq
....................Aacccuguaguuccggauu.........................................................................   17       1          seq
....................uacccuguaguuccggauA.........................................................................   31       1          seq
....................uacccuguaguuccAgauu.........................................................................   3        1          seq
....................uacccuguagCuccggauu.........................................................................   1        1          seq
....................uacccuguagAuccggauuu........................................................................   1        1          seq
....................uacccuguaguuccggauCu........................................................................   1        1          seq
....................uacccuguaguuccggauuG........................................................................   33       1          seq
....................uacccuguaguuAcggauuu........................................................................   4        1          seq
....................Aacccuguaguuccggauuu........................................................................   16       1          seq
....................uacccuguaguuccgUauuu........................................................................   1        1          seq
....................uacccuguaguuccggGuuu........................................................................   2        1          seq
....................uacccuguagGuccggauuu........................................................................   1        1          seq
....................uacccuguaguuccAgauuu........................................................................   2        1          seq
....................uacccAguaguuccggauuu........................................................................   1        1          seq
....................Gacccuguaguuccggauuu........................................................................   1        1          seq
....................uaccUuguaguuccggauuu........................................................................   2        1          seq
....................uacccuguaguucUggauuu........................................................................   1        1          seq
....................uUcccuguaguuccggauuu........................................................................   1        1          seq
....................uacAcuguaguuccggauuu........................................................................   1        1          seq
....................uacccuguagCuccggauuu........................................................................   1        1          seq
....................uacccuguaguuccggauuu........................................................................   2734     0          seq
....................uacccCguaguuccggauuu........................................................................   1        1          seq
....................uacccuguaguucAggauuu........................................................................   2        1          seq
....................uacccuguaguuUcggauuu........................................................................   2        1          seq
....................uaAccuguaguuccggauuu........................................................................   1        1          seq
....................Nacccuguaguuccggauuu........................................................................   2        1          seq
....................uacccuguaguuccggaAuu........................................................................   2        1          seq
....................uacccuguaAuuccggauuu........................................................................   1        1          seq
....................uacccugAaguuccggauuu........................................................................   1        1          seq
....................uacccuguaguuccggauGu........................................................................   7        1          seq
....................uacccuguaguAccggauuu........................................................................   1        1          seq
....................uacccuguaguuccggauAu........................................................................   1        1          seq
....................uacccuguGguuccggauuu........................................................................   1        1          seq
....................uacccuguaguCccggauuug.......................................................................   39       1          seq
....................uacccuguaguuccggUuuug.......................................................................   29       1          seq
....................uacccuguaguuccgUauuug.......................................................................   14       1          seq
....................uNcccuguaguuccggauuug.......................................................................   8        1          seq
....................uacccAguaguuccggauuug.......................................................................   131      1          seq
....................uacccuguGguuccggauuug.......................................................................   56       1          seq
....................uacccuguaguuccggNuuug.......................................................................   2        1          seq
....................uacccuguaguuccgAauuug.......................................................................   37       1          seq
....................uacccNguaguuccggauuug.......................................................................   10       1          seq
....................uacccuguagGuccggauuug.......................................................................   20       1          seq
....................uacccuguagAuccggauuug.......................................................................   149      1          seq
....................uacccuguaguuccggauuuC.......................................................................   25       1          seq
....................uacccuguaguucUggauuug.......................................................................   241      1          seq
....................uacccuguaUuuccggauuug.......................................................................   24       1          seq
....................uacccuguaguuccggauuug.......................................................................   331567   0          seq
....................Nacccuguaguuccggauuug.......................................................................   72       1          seq
....................uacccuguaguuccggaCuug.......................................................................   24       1          seq
....................uacccuguaguuccggauuuA.......................................................................   64       1          seq
....................uacAcuguaguuccggauuug.......................................................................   134      1          seq
....................uacccuguaguuccggaGuug.......................................................................   58       1          seq
....................uacccuguaguuUcggauuug.......................................................................   74       1          seq
....................uacGcuguaguuccggauuug.......................................................................   18       1          seq
....................uacccugNaguuccggauuug.......................................................................   1        1          seq
....................uacccuCuaguuccggauuug.......................................................................   9        1          seq
....................uacccuguaguucGggauuug.......................................................................   26       1          seq
....................uacccuguaguuccggauGug.......................................................................   17       1          seq
....................uacccuguaguuccggaAuug.......................................................................   64       1          seq
....................uacccuAuaguuccggauuug.......................................................................   53       1          seq
....................uCcccuguaguuccggauuug.......................................................................   7        1          seq
```

```
                    Mature                                      Star
cuucagugcuccucaauguuuacccuguaguuccggauuugucuugaaucucagcaaagcaaacaaauucgauuccucgggguuucauguugagugagacuuaucuucuagg
....................uacccGguaguuccggauuug.......................................................................   4      1   seq
....................uaNccuguaguuccggauuug.......................................................................   3      1   seq
....................uacccuguaguuccUgauuug.......................................................................   36     1   seq
....................uacccuguaguuAcggauuug.......................................................................   228    1   seq
....................uacccuguaNuuccggauuug.......................................................................   2      1   seq
....................uacccCguaguuccggauuug.......................................................................   49     1   seq
....................uacccugGaguuccggauuug.......................................................................   9      1   seq
....................uaUccuguaguuccggauuug.......................................................................   23     1   seq
....................uUcccuguaguuccggauuug.......................................................................   25     1   seq
....................uacccugAaguuccggauuug.......................................................................   102    1   seq
....................uaAccuguaguuccggauuug.......................................................................   56     1   seq
....................uacccuguaguGccggauuug.......................................................................   14     1   seq
....................Aacccuguaguuccggauuug.......................................................................   1381   1   seq
....................uacccuguaAuuccggauuug.......................................................................   11     1   seq
....................uacccuguCguuccggauuug.......................................................................   2      1   seq
....................uacccuguaguAccggauuug.......................................................................   101    1   seq
....................uacccuguaguuccAgauuug.......................................................................   432    1   seq
....................Cacccuguaguuccggauuug.......................................................................   47     1   seq
....................uaGccuguaguuccggauuug.......................................................................   6      1   seq
....................uaccGuguaguuccggauuug.......................................................................   17     1   seq
....................uacccuguaguuccggauCug.......................................................................   22     1   seq
....................uacccuguaguuccggauuuU.......................................................................   173    1   seq
....................uGcccuguaguuccggauuug.......................................................................   36     1   seq
....................uacccuguaguuccggauAug.......................................................................   49     1   seq
....................uacccuguaCuuccggauuug.......................................................................   11     1   seq
....................uacccuguUguuccggauuug.......................................................................   14     1   seq
....................uacccuguaguuccCgauuug.......................................................................   4      1   seq
....................uacccuguagCuccggauuug.......................................................................   58     1   seq
....................uacccugCaguuccggauuug.......................................................................   35     1   seq
....................uacccuUuaguuccggauuug.......................................................................   21     1   seq
....................uacccuguaguucAggauuug.......................................................................   100    1   seq
....................uacccuguaguuccggauuAg.......................................................................   71     1   seq
....................uacccuguaguuccggauuGg.......................................................................   45     1   seq
....................uacccuguaguuccggGuuug.......................................................................   46     1   seq
....................uacUcuguaguuccggauuug.......................................................................   106    1   seq
....................uacccuguaguuGcggauuug.......................................................................   14     1   seq
....................uaccAuguaguuccggauuug.......................................................................   66     1   seq
....................uacccuguaguuccgCauuug.......................................................................   13     1   seq
....................uacccuguagNuccggauuug.......................................................................   1      1   seq
....................Gacccuguaguuccggauuug.......................................................................   72     1   seq
....................uacccuguaguuccggauuCg.......................................................................   77     1   seq
....................uaccUuguaguuccggauuug.......................................................................   196    1   seq
....................uacccuguaAuuccggauuugu......................................................................   714    1   seq
....................Aacccuguaguuccggauuugu......................................................................   55144  1   seq
....................uacccugAaguuccggauuugu......................................................................   4192   1   seq
....................uacccCguaguuccggauuugu......................................................................   1985   1   seq
....................uaGccuguaguuccggauuugu......................................................................   364    1   seq
....................uacccuguaguuccggaCuugu......................................................................   1344   1   seq
....................uacccAguaguuccggauuugu......................................................................   4526   1   seq
....................uacccuguaguuccggauuCgu......................................................................   2437   1   seq
....................uacccuguagGuccggauuugu......................................................................   1041   1   seq
....................uacccuguaguuGcggauuugu......................................................................   1023   1   seq
....................uacccuguaguuccggNuuugu......................................................................   19     1   seq
....................uacccuguaNuuccggauuugu......................................................................   59     1   seq
....................uacccuguaguuccggCuuugu......................................................................   41     1   seq
....................uacccuguaguGccggauuugu......................................................................   661    1   seq
....................uacccNguaguuccggauuugu......................................................................   253    1   seq
....................uacccuguaguuAcggauuugu......................................................................   8210   1   seq
....................uacccuguaguuccggauuAgu......................................................................   3034   1   seq
....................uaccGuguaguuccggauuugu......................................................................   799    1   seq
....................uacccuguUguuccggauuugu......................................................................   384    1   seq
....................uacccuguaguNccggauuugu......................................................................   6      1   seq
....................uacccuguaguuccgCauuugu......................................................................   471    1   seq
....................uaNccuguaguuccggauuugu......................................................................   41     1   seq
....................uacccuguaguuccgUauuugu......................................................................   824    1   seq
....................uacccuguaguuccggauuNgu......................................................................   16     1   seq
....................uacccuguaguuccggauuugG......................................................................   12105  1   seq
....................uacccuguaguuccgNauuugu......................................................................   11     1   seq
....................uacccuguaguuccggauCugu......................................................................   1239   1   seq
....................uacccuguaguuccggauuugC......................................................................   1484   1   seq
```

Mature **Star**

cuucagugcuccucaauguuuacccuguaguuccggauuugucuugaaucucagcaaagcaaacaaauucgauuccucgggguuucauguugagugagacuuaucuucuagg			
....................uaccNuguaguuccggauuugu..	1	1	seq
....................uacccugCaguuccggauuugu..	1570	1	seq
....................uacccuguaguuccggauNugu..	14	1	seq
....................uacccugNaguuccggauuugu..	1	1	seq
....................uGcccuguaguuccggauuugu..	1751	1	seq
....................uaccAuguaguuccggauuugu..	2361	1	seq
....................uacccuNuaguuccggauuugu..	6	1	seq
....................uacccuguaguuccggGuuugu..	2092	1	seq
....................uacccuguaguuccggaGuugu..	2851	1	seq
....................uacccuguaUuuccggauuugu..	672	1	seq
....................uacccuguaguuccgAauuugu..	1707	1	seq
....................uacccuguagCuccggauuugu..	1985	1	seq
....................uacccuguaguCccggauuugu..	2162	1	seq
....................uUcccuguaguuccggauuugu..	1008	1	seq
....................uacccuCuaguuccggauuugu..	585	1	seq
....................uacccuguaguuUcggauuugu..	3570	1	seq
....................uNcccuguaguuccggauuugu..	468	1	seq
....................uacccuguaguuccAgauuugu..	19036	1	seq
....................uacccuguagNuccggauuugu..	17	1	seq
....................uacccuguaguuccggauuugu..	13448591	0	seq
....................uacccuguaguucAggauuugu..	3539	1	seq
....................uacAcuguaguuccggauuugu..	4633	1	seq
....................uacccGguaguuccggauuugu..	633	1	seq
....................Cacccuguaguuccggauuugu..	2262	1	seq
....................uaUccuguaguuccggauuugu..	998	1	seq
....................uacccuguaguuccggauuugN..	23	1	seq
....................uacccuguaguuccggauuuAu..	1444	1	seq
....................uacccuguaguuccCgauuugu..	398	1	seq
....................uacccuguaguuccUgauuugu..	1335	1	seq
....................uacccuUuaguuccggauuugu..	698	1	seq
....................uacccuguaguucNggauuugu..	12	1	seq
....................uaccUuguaguuccggauuugu..	8190	1	seq
....................uacccuguaguuccggauAugu..	2388	1	seq
....................uacccuguaguuccggaAuugu..	3379	1	seq
....................uacccuguaguuccggauuuUu..	752	1	seq
....................uacccuguagAuccggauuugu..	6923	1	seq
....................uacccuAuaguuccggauuugu..	1832	1	seq
....................uacccuguaguucGggauuugu..	850	1	seq
....................uacccuguaCuuccggauuugu..	381	1	seq
....................uacccuguaguuccggauGugu..	768	1	seq
....................uacccuguaguuccggauuugA..	8763	1	seq
....................uacccuguaguAccggauuugu..	4110	1	seq
....................uacccugGaguuccggauuugu..	373	1	seq
....................uacccuguaguuccggauuuCu..	749	1	seq
....................Nacccuguaguuccggauuugu..	3504	1	seq
....................Gacccuguaguuccggauuugu..	3400	1	seq
....................uaAccuguaguuccggauuugu..	2397	1	seq
....................uacccuguGguuccggauuugu..	2458	1	seq
....................uCcccuguaguuccggauuugu..	199	1	seq
....................uacccuguaguuccggauuuNu..	63	1	seq
....................uacccuguCguuccggauuugu..	94	1	seq
....................uacccuguaguucUggauuugu..	9833	1	seq
....................uacUcuguaguuccggauuugu..	4582	1	seq
....................uacccuguaguuccggauuGgu..	364	1	seq
....................uacccuguaguuccggUuuugu..	1774	1	seq
....................uacGcuguaguuccggauuugu..	512	1	seq
....................uacccuguNguuccggauuugu..	12	1	seq
....................uacccuguaguuccggauuuguG...	35	1	seq
....................uacccuguaguuccgCauuuguc...	1	1	seq
....................uacccuguGguuccggauuuguc...	1	1	seq
....................uacccuguaguuccggauuuguA...	6733	1	seq
....................uacccugAaguuccggauuuguc...	1	1	seq
....................Aacccuguaguuccggauuuguc...	12	1	seq
....................uacccuguaguuccggauuuguN...	14	1	seq
....................uacccuguaguuccAgauuuguc...	1	1	seq
....................uaAccuguaguuccggauuuguc...	2	1	seq
....................uacccuguaguuccggauuuguU...	30730	1	seq
....................uacccuguaguuAcggauuuguc...	1	1	seq
....................uacccuguaguuccggauuuguc...	1114	0	seq
....................uacccuguaguuccgAauuuguc...	1	1	seq

Mature **Star**

```
cuucagugcuccucaaguuuuacccuguaguuccggauuugucuugaaucucagcaaagcaaacaaauucgauuccucgggguuucauguugagugagacuuaucuucuagg
....................uacccuguaguGccggauuuguc.....................................................................  1     1  seq
....................uacccuguaguuccggauuAguc.....................................................................  1     1  seq
....................uacccuguaguuccggauuugGc.....................................................................  1     1  seq
....................uacccuguaguucUggauuuguc.....................................................................  21    1  seq
....................uacccuguaguuccggauuugAcu....................................................................  1     1  seq
....................uacccuguaguuccggauuugucG....................................................................  3     1  seq
....................uacUcuguaguuccggauuugucu....................................................................  2     1  seq
....................Aacccuguaguuccggauuugucu....................................................................  2     1  seq
....................uacccuguaguuccggauuugucA....................................................................  265   1  seq
....................uacccuguaguuccggaCuugucu....................................................................  1     1  seq
....................uacccuguaAuuccggauuugucu....................................................................  1     1  seq
....................uacccuguaguuccggauuuguGu....................................................................  40    1  seq
....................uacccuguaguuccggauuugucu....................................................................  638   0  seq
....................uaccUuguaguuccggauuugucu....................................................................  1     1  seq
....................uacccuguaguuccggGuuugucu....................................................................  1     1  seq
....................uacccuguaguuccggauuuguAu....................................................................  586   1  seq
....................uacccuguaguuUcggauuugucu....................................................................  1     1  seq
....................uacccuguaguuccggauuuguUu....................................................................  3257  1  seq
....................uacccuguaguuccggauuuguUuu...................................................................  121   1  seq
....................uacccuguaguuccggauuugucuu...................................................................  24    0  seq
....................uacccuguaguuccggauuuguAuu...................................................................  23    1  seq
....................uacccuguaguuccggauuugucuuU..................................................................  2     1  seq
....................uacccuguaguuccggauuugucuuga.................................................................  2     0  seq
....................uacccuguaguuccggauuugucuugaa................................................................  1     0  seq
....................uacccuguaguuccggauuugucuugaau...............................................................  3     0  seq
.....................acccuguaguuccggauuug.......................................................................  63    0  seq
.....................acccAguaguuccggauuug.......................................................................  54    1  seq
.....................acccuguaguAccggauuug.......................................................................  1     1  seq
.....................acccGguaguuccggauuug.......................................................................  1     1  seq
.....................acccuguaguuAcggauuug.......................................................................  15    1  seq
.....................Gcccuguaguuccggauuug.......................................................................  5     1  seq
.....................acAcuguaguuccggauuugu......................................................................  1     1  seq
.....................acccGguaguuccggauuugu......................................................................  48    1  seq
.....................acccuguaguuccAgauuugu......................................................................  2     1  seq
.....................acccCguaguuccggauuugu......................................................................  2     1  seq
.....................Gcccuguaguuccggauuugu......................................................................  69    1  seq
.....................acccuguaguuUcggauuugu......................................................................  1     1  seq
.....................acccuguaguuccggauuugG......................................................................  2     1  seq
.....................aAccuguaguuccggauuugu......................................................................  2     1  seq
.....................acccuguUguuccggauuugu......................................................................  1     1  seq
.....................acccugAaguuccggauuugu......................................................................  2     1  seq
.....................acccuUuaguuccggauuugu......................................................................  1     1  seq
.....................Ccccuguaguuccggauuugu......................................................................  2     1  seq
.....................acccuguaguCccggauuugu......................................................................  1     1  seq
.....................acccuguGguuccggauuugu......................................................................  1     1  seq
.....................acccuguaguuccggauuugu......................................................................  3055  0  seq
.....................acccuguaguucUggauuugu......................................................................  2     1  seq
.....................acccuCuaguuccggauuugu......................................................................  2     1  seq
.....................acccuguagAuccggauuugu......................................................................  1     1  seq
.....................acccAguaguuccggauuugu......................................................................  1505  1  seq
.....................acccuguaguucAggauuugu......................................................................  2     1  seq
.....................Ucccuguaguuccggauuugu......................................................................  33    1  seq
.....................acccuguaguuccCgauuugu......................................................................  1     1  seq
.....................acccuguaguuccggaAuugu......................................................................  2     1  seq
.....................acccuAuaguuccggauuugu......................................................................  1     1  seq
.....................acccuguaguuccggauAugu......................................................................  2     1  seq
.....................acccuguaguuGcggauuugu......................................................................  1     1  seq
.....................acccuguaguuccggauuuUu......................................................................  12    1  seq
.....................acccugCaguuccggauuugu......................................................................  1     1  seq
.....................acccuguaguuAcggauuugu......................................................................  583   1  seq
.....................acccuguaguuccggauuuguU.....................................................................  20    1  seq
.....................acccuguaguuccggauuuguA.....................................................................  3     1  seq
.....................acccAguaguuccggauuuguc.....................................................................  1     1  seq
.....................acccuguaguuccggauuuguc.....................................................................  3     0  seq
.....................acccuguaguuAcggauuuguc.....................................................................  1     1  seq
......................Accuguaguuccggauuug.......................................................................  1     1  seq
......................cccuguaguuccggauuugA......................................................................  1     1  seq
......................Accuguaguuccggauuugu......................................................................  2     1  seq
......................cccugAaguuccggauuugu......................................................................  1     1  seq
......................cccuguaguuccUgauuugu......................................................................  1     1  seq
```

```
                    Mature                                    Star
cuucagugcuccucaauguuuacccuguaguuccggauuugucuugaaucucagcaaagcaaacaaauucgauuccucgggguuucauguugagugagacuuaucuucuagg
......................cccuguaguuccggauuugu......................................................................    148   0   seq
......................cccuguaAuuccggauuugu......................................................................    1     1   seq
......................cccuguaguuccggauuuguU.....................................................................    1     1   seq
.......................ccuguaguuccggauuug.......................................................................    7     0   seq
.......................ccuguaguuccggauuugG......................................................................    1     1   seq
.......................ccuguaguuAcggauuugu......................................................................    2     1   seq
.......................ccuguaguuccAgauuugu......................................................................    2     1   seq
.......................ccuguaguAccggauuugu......................................................................    1     1   seq
.......................Acuguaguuccggauuugu......................................................................    2     1   seq
.......................ccuguaguuccggaAuugu......................................................................    1     1   seq
.......................ccuguagAuccggauuugu......................................................................    1     1   seq
.......................Ucuguaguuccggauuugu......................................................................    5     1   seq
.......................ccuguaguuccggauuugC......................................................................    2     1   seq
.......................ccuguaguucUggauuugu......................................................................    1     1   seq
.......................ccugAaguuccggauuugu......................................................................    1     1   seq
.......................ccuguaguuccggUuuugu......................................................................    1     1   seq
.......................ccuguaguuccggauuCgu......................................................................    1     1   seq
.......................ccuguaguuccggauuugu......................................................................    1199  0   seq
.......................ccuguaguuccggauuuguA.....................................................................    1     1   seq
.......................ccuguaguuccggauuuguU.....................................................................    4     1   seq
.......................ccuguaguuccggauuuguUu....................................................................    20    1   seq
........................cuguaguucUggauuugu......................................................................    1     1   seq
........................Uuguaguuccggauuugu......................................................................    1     1   seq
........................cuguaguuccggauuugu......................................................................    641   0   seq
........................cuguaguuccAgauuugu......................................................................    1     1   seq
........................cuguaguuccggauuuUu......................................................................    1     1   seq
........................cuguaguAccggauuugu......................................................................    1     1   seq
........................cuguaguuAcggauuugu......................................................................    1     1   seq
........................cuguaguuccggauuuguU.....................................................................    2     1   seq
........................cuguaguuccggauuuguc.....................................................................    2     0   seq
..........................................cuugaaucucagcaaagcaaac................................................    3     0   seq
...............................................................caaauucgauuccucgAgguuuca.........................    1     1   seq
...............................................................caaauucgauuccucgggguuuca.........................    2     0   seq
................................................................aaauucgauuccucggggu.............................    1     0   seq
................................................................aaauucgauuccucgggguu............................    8     0   seq
................................................................aaauucgauuccucgggguuG...........................    1     1   seq
................................................................aaauucgauuccucgggguuu...........................    29    0   seq
................................................................aaauucgauuAcucgggguuu...........................    1     1   seq
................................................................aaauucgauuccucgggguuuc..........................    24    0   seq
................................................................aaauucgauuccucgggguuuUa.........................    1     1   seq
................................................................aaauucgauuccucgggguuuca.........................    12    0   seq
```
